# 

**Published:** 1885-02

**Authors:** 


10 cts. a copy. FEB. 1885. $1.00 per Year.

### HALL'S JOURNAL OF HEALTH

ESTABLISHED 1854

Office, 75 & 77 Barclay Street, New York.

Entered as Second-class Matter at the Post-office, New York.

